# Supplementary material for: Microwave imaging of quasi-periodic pulsations at flare current sheet
Source: Nat Commun. 2022 Dec 12;13:7680. doi: 10.1038/s41467-022-35377-0 (PMC9744830; doi:10.1038/s41467-022-35377-0)
Supplement: Supplementary file 1 — Supplementary Information [file 41467_2022_35377_MOESM1_ESM.pdf]

Microwave Imaging of Quasi-periodic Pulsations at Flare Current Sheet

Supplementary Figures

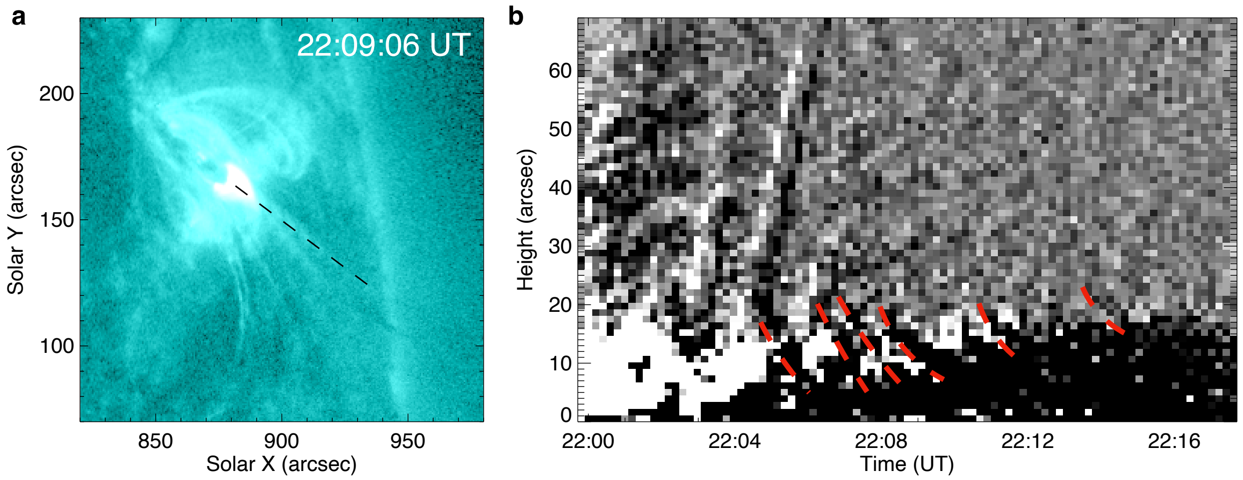

Supplementary Fig. 1: Downflows above cusp-shaped flare arcades. a. AIA 131 Å image at 22:09:06 UT showing the post-flare loops. The black dash line marks the slice for making height-time plot. **b.** Height-time plot of the AIA 131 Å running difference images along the slice as indicated in panel **a**. The trajectories of the downflows are delineated with red dashed curves.

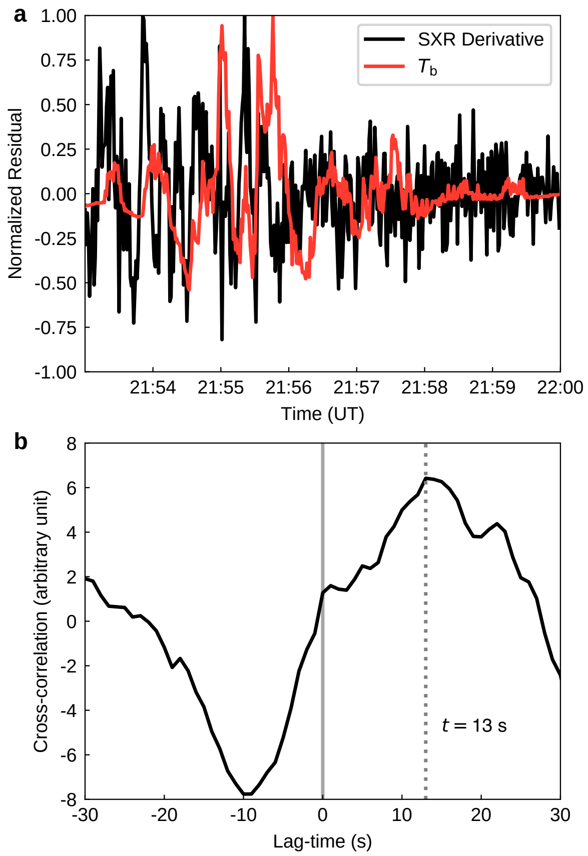

Supplementary Fig. 2: Cross-correlation analysis between brightness temperature variation and time derivative of the SXR flux. a. Normalized and detrended curves of brightness temperature, T_b, of the CS sources at 8.4 GHz (red) and time derivative of SXR flux (black). **b.** Cross-correlation versus the lag-time ranging from -30 to 30 s for the derivative of the SXR flux. The vertical dotted line marks the time when the cross-correlation peaks. The vertical gray solid line marks the reference time ($t = 0$ s).

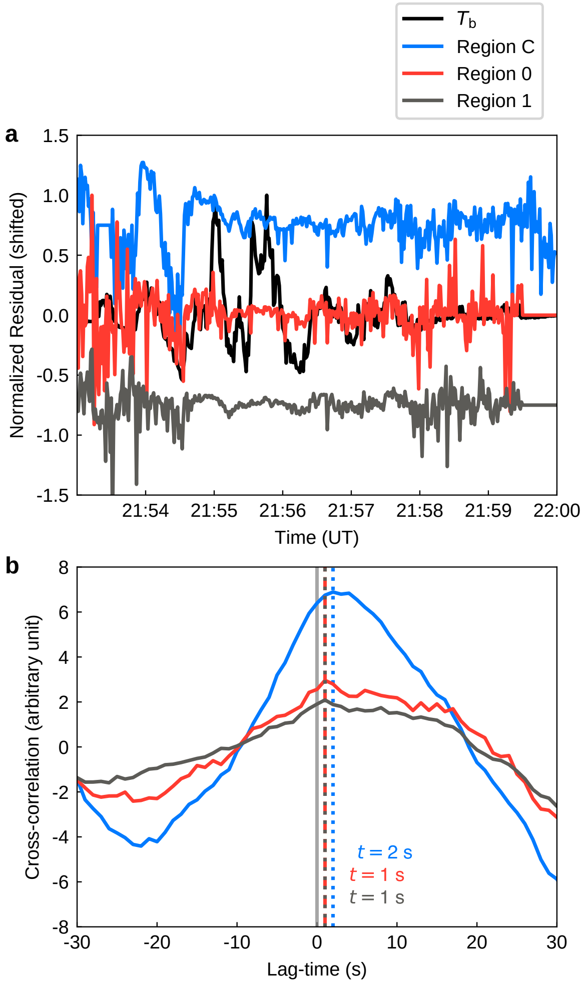

Supplementary Fig. 3: Cross-correlation analyses between brightness temperature and spectral index variations. a. Normalized and detrended curves of T_b of the CS sources at 8.4 GHz (black) and the temporal variations of spectral indices at Region C, 0, and 1 (as indicated in Fig. 4c). **b.** Cross-correlation versus lag-time ranging from -30 to 30 s for spectral indices at Region C, 0, and 1, respectively. The vertical dotted lines mark the time when the cross-correlation peaks, at each Region respectively. The vertical gray solid line marks the reference time ($t = 0$ s).
